# A meta-analysis of resveratrol protects against cerebral ischemia/reperfusion injury: Evidence from rats studies and insight into molecular mechanisms

**DOI:** 10.3389/fphar.2022.988836

**Published:** 2022-10-05

**Authors:** Ruirui Xue, Shuang Gao, Yayun Zhang, Xuejun Cui, Wen Mo, Jinhai Xu, Min Yao

**Affiliations:** ^1^ Department of Orthopedics and Traumatology, Longhua Hospital, Shanghai University of Traditional Chinese Medicine, Shanghai, China; ^2^ Department of Geriatrics, Longhua Hospital, Shanghai University of Traditional Chinese Medicine, Shanghai, China; ^3^ Spine Disease Institute, Longhua Hospital, Shanghai University of Traditional Chinese Medicine, Shanghai, China

**Keywords:** cerebral ischemia/reperfusion injury, resveratrol, meta-analysis, antioxidation, neuroprotective

## Abstract

**Objective:** To evaluate the neuroprotective effect of resveratrol (RES) in rat models of cerebral ischemia/reperfusion (I/R) injury.

**Data sources:** PubMed, Embase, MEDLINE, Cochrane Library, and Chinese databases were searched from their inception dates to July 2022. No language restriction was used in the literature search.

**Date Selection:** Studies were selected that RES were used to treat cerebral I/R injury *in vivo*. Two reviewers conducted literature screening, data extraction and methodological quality assessment independently.

**Outcome measures:** Cerebral infarct volume was included as primary outcome. The secondary outcomes included cerebral water content and neurological deficit scores. Malondialdehyde (MDA) and superoxide dismutase (SOD) were used to evaluate oxidative stress during medication.

**Results:** A total of 41 studies were included, and only a few of them the methodological quality was relatively low. Compared with the control group, RES significantly reduced the cerebral infarct volume (29 studies, standard mean difference (SMD) = −2.88 [−3.23 to −2.53], *p <* 0.00001) and brain water content (nine studies, MD = −9.49 [−13.58 to −5.40], *p <* 0.00001) after cerebral I/R injury, then neurological function was improved (15 studies, SMD = −1.96 [−2.26 to −1.65], *p <* 0.00001). The MDA level (six studies, SMD = −8.97 [−13.60 to −4.34], *p* = 0.0001) was decreased notably after treatment of RES, while the SOD level (five studies, SMD = 3.13 [−0.16 to 6.43], *p* = 0.06) was increased unsatisfactory. Consistently, subgroup analysis of cerebral infarct volume suggested that the optimal therapeutic dose is 30 mg/kg (eight studies, SMD = −5.83 [−7.63 to −4.04], *p* < 0.00001). Meanwhile, 60 min of occlusion (three studies, SMD = −10.89 [−16.35 to −5.42], *p <* 0.0001) could get maximum benefit from compared with 90 min and 120 min of occlusion. On the other hand, the publication bias cannot be ignored. The pharmacological mechanisms of RES on cerebral I/R injury models as reported have be summarized, which can be used for reference by researchers to further plan their future experiments.

**Conclusion:** RES might have a good neuroprotective effect on cerebral I/R injury in rats, then 30 mg/kg RES may be the optimal dose for treatment, and early administration of RES should be more neuroprotective. Also it need to be further verified through exploration of dose effect relationship, or delay administration or not.

## Introduction

Ischemic stroke, a severe threat to human health, is regarded as a major cause of death and disability around the world, accounting for 87% of all strokes and placing a heavy economic burden on families and society (Virani et al., 2021). The incidence of ischemic stroke in young people has been rising since the 1980s with a high mortality rate, make it no longer a disease exclusive to the elderly (Putaala, 2020). The cost of per patient every year was reported as $59,900 in the United States (Strilciuc et al., 2021). Complications (eg, epilepsy, pain, depression, and cognitive problems), further aggravate the economic burden, are the top concern in stroke survivors. Recent evidence suggests that it is feasible to provide appropriate level of stroke care and preventive interventions in low-income or middle-income countries (Yan et al., 2016). In this case, there is an increasing demand for treatments with high cure rates and good long-term outcomes.

In general, ischemic stroke refers to a condition that occurs when blood flow to the brain is sharply reduced, depriving the tissue of blood and oxygen, resulting in the death of nerve cells and cerebral infarction. Timely and effective revascularization after ischemic stroke would be particularly significant (Candelario-Jalil, 2009). The fundamental technique for the treatment of acute ischemic stroke within 4.5 h refers to intravenous thrombolysis or mechanical thrombectomy within 24 h, since it could ameliorate functional outcomes to some extent (Tsivgoulis et al., 2017; Mendelson and Prabhakaran, 2021). Nevertheless, the limited therapeutic time window and complex contraindications limit its application. Meanwhile, damaged brain tissue and nerve function will be further aggravated following reperfusion, the whole process, from ischemic to reperfusion, named cerebral I/R injury. It is deemed as an extremely complex pathological process, which might lead to a series of pathological reactions containing glutamate excitotoxicity, oxidative stress, calcium overload, inflammation, apoptosis and autophagy (Sun et al., 2020; Zhang et al., 2020; Liu J. et al., 2021; Mandalaneni et al., 2022). In terms of pathophysiology, there are a number of potential targets for treatment in cerebral I/R injury. To be specific, therapeutic targets normally incorporate attenuating the excess reactive oxygen species (ROS), mitigating the effects of inflammatory cascades, along with inhibiting leukocyte activation and platelet recruitment (Mandalaneni et al., 2022). Despite the fact that, a large number of researches and experiments have been done in the field of neuronal, endothelial, and glial protective therapies, the research on therapeutic strategies for cerebral I/R injury is still in its infancy (Matei et al., 2020). Undeniably, drugs or means with potent free radical-scavenging properties have become important targets in the treatment of cerebral I/R injury (Bao et al., 2018).

Resveratrol (3,4,5-trihydroxy-trans-stilbene), a natural phytoalexin polyphenol, traditional plants contained have been used effectively in traditional Chinese medicine for over 2000 years, and the most abundant Chinese medicine involving resveratrol is *Polygonum Cuspidatum (* *Gambini et al., 2015* *)*. Up till now, resveratrol has been used to treat cancer, inflammation, diabetes, myocardial I/R injury, and other diseases (Mao et al., 2019; Ashrafizadeh et al., 2021; Hoca et al., 2021; Meng et al., 2021). According to some researches, the neuroprotective effects of resveratrol have been demonstrated through its antioxidant and anti-inflammatory abilities (Rocha-González et al., 2008; Ro et al., 2021). Numerous animal experiments have been conducted to evaluate the neuroprotective effect of resveratrol against cerebral I/R injury. Recent meta-analysis had demonstrated neuroprotective effects of resveratrol treatment in ischemic stroke rodent models with English language studies restriction (Liu X. et al., 2021). Therefore, we conducted a comprehensive systematic review and meta-analysis of the neuroprotective effect of resveratrol from rats studies and insight into molecular mechanisms, based on studies without language restriction to avoid a degree of selection bias, and only the data related to rats was included to avoid the heterogeneity caused by the different species of rats and mouse (Bonthuis et al., 2010; Vesterinen et al., 2014).

## Data and methods

### Search strategy

We scientifically searched the PubMed, Embase, Web of Science, China National Knowledge Infrastructure, Wanfang, China Biology Medicine databases, and Cochrane databases for resveratrol in rats model on cerebral I/R injury published from the inception to July 2022, without any language restriction. The following terms were used for the search: (“cerebral ischemia/reperfusion injury” OR “ischemia-reperfusion injury” OR “brain ischemia/reperfusion injury” OR “I/R injury” OR “stroke” OR “cerebral ischemia” OR “cerebral ischemic”) AND (“resveratrol” OR “RES” “cis-resveratrol” OR “trans-resveratrol” OR “3,4,5-trihydroxy-trans-stilbene” OR “resveratrol-3-sulfate” OR “SRT 501” OR “SRT501” OR “SRT-501”) In addition, the reference of review articles, meeting abstracts, and comments for additional citations were also scrutinized.

### Inclusion and exclusion criteria

Studies that met the following inclusion criteria were included: *1*) Types of included animals: rats as experimental animal involving any size, weight, and I/R modeling method. *2*) Types of treatment: all types of resveratrol treatment, involving studies with combined treatments, then derivative of resveratrol was excluded. With no limitations on administration, formulation or dosage. *3*) Primary outcome: brain edema and the infarct size can be used as indicators of the brain injury process. The infarct size was determined by triphenyl tetrazolium chloride (TTC) staining. The following equation was used to calculate the water content of the brain: H_2_O (%) = (wet weight (WW) − dry weight (DW))/WW×100%. Secondary outcomes included behavioral assessment, which was evaluated by neurological deficit scores, as well as biochemical examination of the peroxidation index. No language, publication date, or publication status restrictions were imposed.

The exclusion criteria are as follows: *1*) Conference papers and publications of abstracts only that lacked quantitative data information. *2*) Repeated publications. *3*) All clinical case reports and solely *in vitro* studies.

### Data extraction

The two reviewers independently extracted data from the included studies and resolved the differences through consensus. The following information extraction of each study is summarized: *1*) The characteristics of the study (e.g., authors name, country, year of publication, including animals, number, modeling method, and duration of I/R injury), *2*) Intervention methods (e.g., route of administration, dosage, type of vehicle, and time of treatment), *3*) Data related to the cerebral infarct volume, brain water content, neurological deficit scores, malondialdehyde (MDA) and superoxide dismutase (SOD). The measured outcomes as the mean ± standard deviation (SD). For data not described numerically in the text, the values in the graph were estimated using commercial software (GetData Graph Digitizer 2.25; download from http://getdata-graph-digitizer.com).

### Quality assessment

The Collaborative Approach to Meta Analysis and Review of Animal Data From Experimental Stroke (CAMARADES) 10-item checklist were used to assess the quality and design of the studies by two independent reviewers (Macleod et al., 2004). The CAMARADES 10-item checklist include the following list: *1*) peer-reviewed journal; *2*) temperature control; *3*) animals were randomly allocated; *4*) blind established model; *5*) blinded outcome assessment; *6*) anesthetics used without marked intrinsic neuroprotective properties; *7*) animal model (diabetic, advanced age or hypertensive); *8*) calculation of sample size; *9*) statement of compliance with animal welfare regulations; *10*) possible conflicts of interest.

### Statistical analysis

Meta-analysis was carried out using Review Manager, version 5.4 (downloaded from https://training.cochrane.org/online-learning/core-software-cochrane-reviews/revman). All the data of resveratrol were gathered together for comparison with the control group. The reported results and the continuous variables were expressed as mean difference (MD) or standardized mean difference (SMD), if at least five studies, the data were pooled. Data about the cerebral infarct volume, brain water content, neurological deficit scores, MDA and SOD were analyzed. The effect size was assessed using MD when the unit of measurement is the same, on the contrary, the SMD was used. Heterogeneity was tested using the chi-square test: *p* < 0.1 indicated heterogeneity, while *p* > 0.1 indicated no heterogeneity. At the same time, the I^2^ statistics were used to assess heterogeneity. When the heterogeneity between studies was low (I^2^ < 50%), the size of the pooled effect was estimated using a fixed-effects model, otherwise a random-effects model was used. Differences were considered statistically significant when *p* < 0.05. The funnel plot test was used to check the meta-analysis publication bias, if more than 10 studies included in the outcome.

## Results

### Search strategy

**FIGURE 1:**
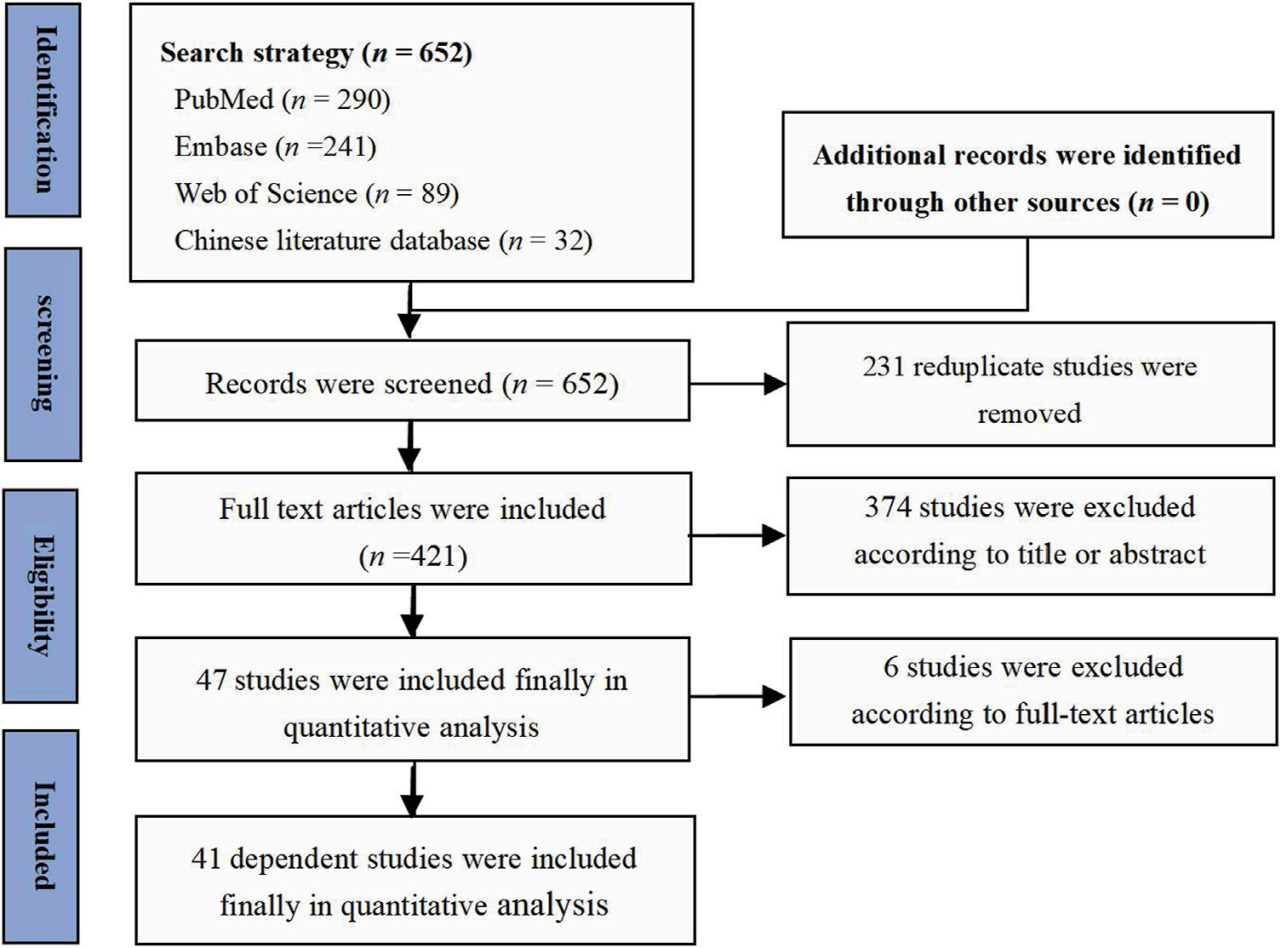
PRISMA flow diagram for review and selection process.

Of the 652 articles found in the initial search strategy, 231 similar and duplicate studies were removed. The remaining 421 records were evaluated in more detail. As a result, a total of 41 articles (Sinha et al., 2002; Shigematsu et al., 2003; Liu et al., 2007; Tsai et al., 2007; Della-Morte et al., 2009; Yousuf et al., 2009; Li et al., 2010; Li et al., 2011; Ren et al., 2011; Simao et al., 2011; Li et al., 2012; Simao et al., 2012; Lin et al., 2013; Simao et al., 2013; Saleh et al., 2014; Fang et al., 2015; Girbovan and Plamondon, 2015; Kizmazoglu et al., 2015; Li et al., 2015; Wei et al., 2015; Girbovan et al., 2016; Li et al., 2016; Shi et al., 2016; Wan et al., 2016; Yang et al., 2016; Dera, 2017; He et al., 2017; Chang et al., 2018; Hou et al., 2018; Liu et al., 2018; Xu et al., 2018; Hong et al., 2019; Lei et al., 2019; Serra et al., 2019; Yan et al., 2019; Zhao et al., 2019; Gutierrez Aguilar et al., 2020; Lu et al., 2020; Pineda-Ramirez et al., 2020; Teertam et al., 2020; Zhu et al., 2022) met our selection criteria. The basic characteristics of the included studies are shown in Table 1. Of the 41 full publications that met the predetermined inclusion criteria, 40 were published in English and one was published in Chinese (Liu et al., 2007) (Figure 1 and Table 1).

**TABLE 1:** Description of the characteristics of studies included in this review.

Study	Animals	I/R	No. of animals	Groups	Administration time	Therapeutic outcome
Zhu H 2022 (China) (Zhu et al., 2022)	Male SD rats (250–300 g)	MCAO 120 min 37°C	4/4/4/4	A. Sham	7 days before I/R	Cerebral infarct volume
				B. MCAO		
				C. MCAO + DMSO		
				D. MCAO + RES (30 mg/kg, *i.p.*)		
Gutiérrez Aguilar GF 2020 (Mexico)	Male Wistar rats (280–350 g)	MCAO 120 min 37°C	—	A. Control	At the onset of reperfusion	Cerebral infarct volume
				B. MCAO + vehicle		
				C. MCAO + RES (1.9 mg/kg, *i.p.*)		
Teertam SK 2020 (India)	Male SD rats (250–300 g)	MCAO 60 min	3/3/3	A. Sham	Prior 30 min of reperfusion	Cerebral infarct volume, neurological deficit scores (0–4)
				B. MCAO		
				C. MCAO + RES (20 mg/kg, *i.p.*)		
Lu X 2020 (China)	Male SD rats (250–300 g)	MCAO 120 min 37 ± 0.5°C	5/4/5/5/4/4	A. Sham	—	Cerebral infarct volume, neurological deficit scores (1–14)
				B. Empty NPs		
				C.RES-NPs (1 mg/kg, *i.p.*)		
				D. RES-NPs (5 mg/kg, *i.p.*)		
				E. RES-NPs (10 mg/kg, *i.p.*)		
				F. RES-Sol (10 mg/kg, *i.p.*)		
Pineda-Ramírez N 2020 (Mexico)	Male Wistar rats (280–350 g)	MCAO 120 min 37°C	6/6/6/6	A. Sham + vehicle	At the onset of reperfusion	Cerebral infarct volume
				B. MCAO + vehicle		
				C. MCAO + RES (1.8 mg/kg *i.v.*)		
				D. MCAO + RES (1.8 mg/kg i.v.) + Compound C (2 mg/kg i.v.)		
Hong G 2019 (China)	SD rats (160–220 g)	BCCAO 120 min	10/10/10/10/10	A. Sham	7 days before I/R	Cerebral infarct volume, cerebral water content
				B. I/R		
				C. IPC		
				D. I/R + RES (20 mg/kg *i.p.*)		
				E. IPC + RES (20 mg/kg *i.p.*)		
Zhao R 2019 (China)	Male SD rats (300–330 g)	MCAO 90 min 37°C	8/8	A. MCAO + vehicle	20 days after I/R	Cerebral infarct volume, cerebral water content, open field tests
				MCAO + RES (10 mg/kg *i.v.gtt*)		
				B		
Lei JR 2019 (China)	SD rats (250–300 g)	MCAO 120 min 37 ± 0.5°C	30/36/30/36	A. Sham	2 h after I/R	Cerebral infarct volume, cerebral water content, neurological deficit scores (4-point)
				B. MCAO + vehicle		
				C. MCAO + RES (10 mg/kg *i.p.*)		
				D. MCAO + RES (100 mg/kg *i.p.*)		
Yan Y 2019 (China)	Male SD rats (160–220 g)	BCCAO 120 min	10/10/10/10/10	A. Sham + vehicle	7 days before I/R	Cerebral infarct size
				B. IR + vehicle		
				C. IPC + vehicle		
				D. IR + RES (20 mg/kg *i.p.*)		
				E. IPC + RES (20 mg/kg *i.p.*)		
Serra MP 2019 (Italy)	male Wistar rats (210 ± 20 g)	BCCAO 30 min		A.Sham + vehicle	**—**	**/-**
				B. Sham + RES (40 mg, *i.g.*)		
				C.BCCAO + vehicle (0.3 ml oil, *i.g.*)		
				D. BCCAO + RES (40 mg, *i.*)		
Hou Y 2018 (China)	Male SD rats (230–270 g)	MCAO 120 min	25/25/25/25/25	A. Sham + vehicle	For 7 days before I/R	Cerebral infarct volume, neurological deficit scores (5-point system)
				B. MCAO + vehicle		
				C. MCAO + RES (30 mg/kg *i.p.*)		
Liu Y 2018 (China)	Male SD rats (200–220 g)	MCAO 90 min 37 ± 0.5°C	15/15/15/15	A. MCAO + vehicle	7 days before I/R	Cerebral infarct volume, mNSS (0–18)
				B. MCAO + RES (50 mg/kg *i.p.*)		
				C.MCAO + ROS (10 mg/kg *i.g.*)		
				D.MCAO + RES (50 mg/kg *i.p.*) +ROS		
Xu H 2018 (China)	Male SD rats (250–300 g)	MCAO 120 min 37 ± 0.5°C	5/5/5/5/5/5	A. MCAO + vehicle	After 2 h occlusion	Cerebral infarct volume, Neurologic Function Scoring System (0–14)
				B. MCAO + Empty-NPs		
				C.MCAO + RES-NPs (5 mg/kg *i.v.*)		
				D.MCAO + RES-NPs (10 mg/kg *i.v.*)		
				E. MCAO + RES-NPs (20 mg/kg *i.v.*)		
				F. MCAO + RES-NPs (40 mg/kg *i.v.*)		
Chang C 2018 (China)	SD rats (260–300 g)	MCAO 120 min		A. Sham	7 days before I/R	Open-field and closed-field test and Morris water maze test
				B. MCAO		
				C.MCAO + RES (20 mg/kg *i.p.*)		
				D.MCAO + vehicl		
He Q 2017 (China)	Male SD rats (250–280 g)	MCAO 60 min		A. Sham	At the onset of I/R	Cerebral infarct volume, brain water content, neurological deficit scores (0–5)
				B. MCAO		
				C. MCAO + vehicle		
				D. MCAO + RES (100 mg/kg *i.p.*)		
AI Dera H 2017 (Kingdom of Saudi Arabia)	Male Wistar rats(230 ± 10 g)	BCCAO 40 min	12/12/12	A. Sham + vehicle	30 days before I/R and 7 days after I/R	Cerebral infarction volume
				B. I/R + vehicle		
				C. I/R + RES (20 mg/kg *i.p.*)		
Wan D 2016 (China)	Male SD rats (280 ± 20 g)	MCAO 120 min	10/10/10/10	A. Sham	5 days before I/R	Cerebral infarct volume, neurological deficit scores (0–4)
				B. MCAO		
				C. MCAO + vehicle		
				D. MCAO + RES (20 mg/kg *i.p.*)		
				E. MCAO + PDE inhibitor		
Yang HN 2016 (China)	Male SD rats (110–140 g)	MCAO 90 min 37 ± 0.5°C	15/15	A. MCAO + vehicle	7 days before I/R	Neurological deficit scores (the 21-point behavioral scale)
				B. MCAO + RES (50 mg/kg *i.p.*)		
Shi N 2016 (China)	Male SD rats (200 ± 20 g)	MCAO 120 min	24/24/24/24/24	A. Sham	21 days after MCAO	Neurological deficit scores (the method of Longa et al.) (0–4)
				B. Model		
				C. MCAO + rehabilitation training		
				D. MCAO + RES (30 mg/kg *i.p.*)		
				E. MCAO + Rehabilitation + RES (30 mg/kg *i.p.*)		
Li Z 2016 (China)	Male SD rats (220–250 g)	MCAO 90 min 37°C		A. Sham + vehicle	1, 4, 6, 12, or 24 h before MCAO	Cerebral infarct volume, the Morris water maze test
				B. Sham + RES (30 mg/kg *i.p.*)		
				C. MCAO + vehicle		
				D. MCAO + RES (30 mg/kg *i.p.*)		
Kizmazoglu C 2015 (Turkey)	Female SD rats (250–300 g)	BCCAO 30 min 37 ± 0.5°C	10/10/10/10/10	A. Sham	after I/R	**—**
				B. I/R		
				C. I/R + RES (20 mg/kg *i.p.*)		
				D. I/R + RES (40 mg/kg *i.p.*)		
				E. I/R + DMSO		
Fang LQ 2015 (China)	Male SD rats (250–300 g)	MCAO 120 min 37°C	13/14/13	A. Sham + vehicle	4 days after I/R	Cerebral infarct volume, brain water content, neurological deficit scores (0–4)
				B. MCAO + vehicle		
				C.MCAO + RES (30 mg/kg *i.p.*)		
Li WN 2015 (China)	Male SD rats (250–300 g)	MCAO 90 min	10/10/10/10	A. Sham + vehicle	10 min before I/R, 0 h, 20 h after I/R	Cerebral infarct volume, brain water content, neurological deficit scores (0–4)
				B. MCAO + vehicle		
				C. MCAO + edaravone		
				D. MCAO+RES (20 mg/kg, *i.p.*)		
Girbovan C 2015 (Canada) (1)	Male Wistar rats(325–375 g)	4-VO 37 ± 0.5°C	7/6/8/8/6	A. Ischemia + vehicle	Twenty-one days before the surgery	**—**
				B. Sham + vehicle (*i.p.*)		
				C. Ischemia + RES (1 mg/kg *i.p.*)		
				D. Ischemia + RES (10 mg/kg *i.p.*)		
				E. Sham + RES (10 mg/kg *i.p.*)		
Girbovan C 2015 (Canada) (2)	Male Wistar rats(325–375 g)	4-VO 37 ± 0.5°C	7,10/6,10/8,12/8,11/6,10	A. Ischemia + vehicle (*i.p.*)	Twenty-one days before the surgery	CA1 neuronal density, spatial memory performance (Morris water maze testing, place task, probe trial, platform-switched task, visual acuity task)
				B. Sham + vehicle (*i.p.*)		
				C. schemia + RES (1 mg/kg *i.p.*)		
				D. Ischemia + RES (10 mg/kg *i.p.*)		
				E. Sham + RES (10 mg/kg *i.p.*)		
Saleh MC 2014(Canada)	Male SD rats (250–350 g)	MCAO 30 min 37 ± 1°C	5/5/6/5/5/5	A. MCAO + RES (2 × 10^–3^, 1 ml/kg *i.v.*)	30 min prior to the onset of MCAO	Cerebral infarct volume
				B. MCAO + RES (2 × 10^–4^, 1 ml/kg *i.v.*)		
				C. MCAO + RES (2 × 10^–5^, 1 ml/kg *i.v.*)		
				D. MCAO + RES (2 × 10^–6^, 1 ml/kg *i.v.*)		
				E. MCAO + RES (2 × 10^–7^, 1 ml/kg *i.v.*)		
				F. MCAO + vehicle (1 ml/kg *i.v.*)		
Wei H 2014 (China)	Male SD rats (260 ± 20 g)	MCAO 120 min 37 ± 0.5°C	7/7/7/7	A. Sham	At the onset of I/R/15 min before I/R	Cerebral infarct volume, brain water content, neurological deficit scores (a score system of Garcia) (0–18)
				B. MCAO		
				C. MCAO + vehicle		
				D. MCAO + RES (50 mg/kg *i.p.*)		
				F. MCAO + RES + IgG		
				G. MCAO + RES + neutralized		
Lin Y 2013 (China)	Male SD rats (200–250 g)	MCAO 120 min 36.5°C–7°C	12/12/12/12	A. Sham + DMSO	7 days before I/R	Cerebral infarct volume, neurological deficit scores (0–4)
				B. MCAO + DMSO		
				C. MCAO + RES (200 mg/kg, *i.p.*)		
				D. MCAO + PD98059		
Simão F 2013 (Brazil)	Male Wistar rats (290–330 g)	4-VO 37 ± 0.5°C	8/8/8/8	A. Sham	7 days before ischemia	**—**
				B. Sham + RES (30 mg/kg *i.p.*)		
				C. ischemia + vehicle (*i.p.*)		
				D. ischemia + RES (30 mg/kg *i.p.*)		
Li Z 2012 (China)	Male SD rats (220–250 g)	MCAO 90 min 37 ± 0.5°C	38/42/45/25	A. Sham + vehicle	3 h after I/R and lasting for 4 days	Cerebral infarct volume
				B. MCAO + vehicle		
				MCAO + RES (30 mg/kg *i.p.*)		
				D. Sham + RES (30 mg/kg *i.p.*)		
Simão F 2012 (Brazil)	Male Wistar rats (290–330 g)	4-VO 37 ± 0.5°C		A. Sham	7 days before ischemia	**—**
				B. Sham + RES (30 mg/kg *i.p.*)		
				C. ischemia + vehicle (*i.p.*)		
				D. ischemia + RES (30 mg/kg *i.p.*)		
Simão F 2011 (Brazil)	Male Wistar rats (290–330 g)	4-VO 37 ± 0.5°C	7/7/7/7	A. Sham + vehicle	7 days before ischemia	**—**
				B. Sham + RES (30 mg/kg *i.p.*)		
				C. ischemia + vehicle		
				D. ischemia + RES (30 mg/kg i.p.)		
Ren JW 2011 (China)	Male SD rats (230–280 g)	MCAO 120 min 37 ± 0.5°C	24/24/24/24	A. Sham + vehicle	7 days before surgery and 30 min before ischemia	Cerebral infarct volume, brain water content, neurological deficit scores (0–4)
				B. MCAO + vehicle		
				C. MCAO + RES (15 mg/kg *i.p.*)		
				D. MCAO + RES (30 mg/kg *i.p.*)		
Li H 2011 (China)	Male SD rats (300 ± 50 g)	MCAO 120 min 37 ± 0.5°C	6/8/8	A. Sham + vehicle	7 days before surgery and 60 min before ischemia	Cerebral infarct volume, neurological deficit scores (0–4)
				B. MCAO + vehicle		
				C. MCAO+RES (30 mg/kg *i.p.*)		
Li C 2010 (China)	Male SD rats (300 ± 50 g)	MCAO 120 min 37 ± 0.5°C	6/8/8	A. Sham + vehicle	7 days before surgery and 60 min before ischemia	Cerebral infarct volume, neurological deficit scores (0–4)
				B. MCAO + vehicle		
				C. MCAO+RES (30 mg/kg *i.p.*)		
Yousuf S 2009 (India) (Yousuf et al., 2009)	Male Wistar rats (300–350 g)	MCAO 120 min 37 ± 0.5°C	8/8/8	A. Sham + vehicle	15 min pre-occlusion and at the time of reperfusion	Cerebral infarct volume, neurological deficit scores (the method of Bederson et al.) (0–4)
				B. MCAO + vehicle		
				C. MCAO+RES (10^–7^ g/kg *i.v.*)		
Della-Morte D2009(USA) (Della-Morte et al., 2009)	SD rats (250–350 g)	ACA 8 min 37°C	6/10/6/8/8/8	A. Sham	48 h before I/R	—
				B. ACA + saline		
				C. IPC + ACA		
				D. ACA + RES (10 mg/kg *i.p.*)		
				E. ACA + RES (50 mg/kg *i.p.*)		
				F. ACA + RES (100 mg/kg *i.p.*)		
Tsai SK 2007 (China)	Male Long-Evans rats (250–320 g)	MCAO 60 min 37 ± 0.5°C	7/12/7/7/10/9/11/9/9	A. Sham	—	Cerebral infarct volume
				B. MCAO + vehicle		
				C. MCAO + RES (0.01 μg/kg *i.v.gtt*)		
				D. MCAO + RES (0.1 μg/kg *i.v.gtt*)		
				E. MCAO + RES (1 μg/kg *i.v.gtt*)		
Liu YG 2007 (China)	Male SD rats (250–300 g)	MCAO 120 min		A. Sham + vehicle	7 days before I/R	Cerebral infarct volume, neurological deficit scores (the method of Longa et al.) (0–4)
				B. MCAO + vehicle		
				C. MCAO + Nimodipine		
				D. MCAO + RES (10 mg/kg *i.g.*)		
				E. MCAO + RES (20 mg/kg *i.g.*)		
				F. MCAO + RES (40 mg/kg *i.g.*)		
Shigematsu S 2003 (USA)	Male SD rats (200–250 g)	ligating the superior mesenteric artery and vein	8/6/6	A. I/R+ untreated	**—**	**—**
				B. I/R + RES (*i.p.*)		
				C. I/R + SOD (30 mg/kg *i.p.*)		
Sinha K 2002 (India)	Male Wistar rats (200–250 g)	MCAO 120 min 37°C	10/16/16	A. Sham	21 days before I/R	Cerebral infarct volume, grip test, rota rod, locomotor activity
				B. MCAO + vehicle (*i.p.*)		
				C.MCAO + trans resveratrol (20 mg/kg *i.p.*)		

BCCAO, the bilateral common carotid arteries occlusion; DMSO, dimethyl sulphoxide; 4-VO, the four-vessel occlusion model; *i*.*g*.,intragastric gavage; *i.p.*, intraperitoneal; *i.v.*, intravenous injection; *i.v.gtt*, intravenously guttae; I/R, ischemia/reperfusion; IPC, Ischemic Preconditioning; L-NIO, L-N5-(1-iminoethyl)-ornithine; L-NAME, N-nitro-L-arginine methyl ester; MCAO, middle cerebral artery occlusion; NPs, nanoparticles; PDE, phosphodiesterase; PD98059, a potent and selective cell permeable inhibitor of MAP kinase kinase; RES, resveratrol; ROS, rosuvastatin; mNSS, the modified neurological severity scores; SD, Sprague-Dawley; SOD, superoxide dismutase.

### Characteristics of the studies

In consequence, the majority of these studies were conducted in China (*n* = 25), with the remaining in the Brazil (*n* = 3), Canada (*n* = 3), India (*n* = 3), Turkey (*n* = 2), Mexico (*n* = 2), USA (*n* = 2), Italy (*n* = 1) and Kingdom of Saudi Arabia (*n* = 1). The sample size of the included studies ranged from 9 to 150. Sprague-Dawley (SD) rats were used in 29 studies, and male Wistar rats were used in 11 studies (Sinha et al., 2002; Yousuf et al., 2009; Simao et al., 2011; Simao et al., 2012; Simao et al., 2013; Girbovan and Plamondon, 2015; Girbovan et al., 2016; Dera, 2017; Serra et al., 2019; Gutierrez Aguilar et al., 2020; Pineda-Ramirez et al., 2020), thus male Long-Evans rats were used in one another study (Tsai et al., 2007). Male SD rats were clearly identified in 24 studies, and one was female (Kizmazoglu et al., 2015). Whereas four studies (Della-Morte et al., 2009; Chang et al., 2018; Hong et al., 2019; Lei et al., 2019) did not determine whether the SD rats were male or female. Cerebral I/R injury was achieved by middle cerebral artery occlusion reperfusion model in 29 studies or bilateral common carotid arteries occlusion reperfusion model in five studies (Kizmazoglu et al., 2015; Dera, 2017; Hong et al., 2019; Serra et al., 2019; Yan et al., 2019). Besides, there were five researches (Simao et al., 2011; Simao et al., 2012; Simao et al., 2013; Girbovan and Plamondon, 2015; Girbovan et al., 2016) adopted the four-vessel occlusion (4-VO) model and one (Della-Morte et al., 2009) used model of asphyxial cardiac arrest (ACA).

### Risk of bias within studies

The included literature was assessed for risk bias, as shown in Table 2, the quality score ranged from 3 to 7. A total of 25 studies got quality assessment score ≥6, which meant high quality of methodology. All studies were published in peer-reviewed journal, of which 28 studies illustrate the control of temperature. No study described sample-size calculation and allocation concealment, on the contrary, all of them involve blind established model. 22 studies reported randomization, and 14 studies reported potential conflicts of interest. 17 studies reported blinded assessment of outcome. 38 studies stated they were in compliance with animal welfare laws. 38 studies pointed out anesthetics used without marked intrinsic neuroprotective properties. Only one study (Dera, 2017) elaborated on inclusion and exclusion criteria, whereas six studies (Ren et al., 2011; Lin et al., 2013; Li et al., 2015; Shi et al., 2016; He et al., 2017; Liu et al., 2018) were clearly proposed to screen according to neurological function score.

**TABLE 2:** Risk of bias summary.

Study	1	2	3	4	5	6	7	8	9	10	Score
Zhu H 2022	+	+	?	?	?	+	?	?	+	+	5
Gutiérrez Aguilar GF 2020	+	+	?	+	?	+	?	?	+	+	6
Teertam SK 2020	+	?	+	+	?	+	?	?	+	+	6
Lu X 2020	+	+	?	+	+	+	?	?	+	+	7
Pineda-Ramírez N 2020	+	+	+	+	?	?	?	?	+	?	5
Hong G 2019	+	?	?	+	?	+	?	?	+	+	5
Zhao R 2019	+	+	+	+	?	+	?	?	+	+	7
Lei JR 2019	+	+	?	+	?	+	?	?	+	+	6
Yan Y 2019	+	?	?	+	+	+	?	?	+	+	6
Serra MP 2019	+	?	+	+	+	+	?	?	+	+	7
Hou Y 2018	+	?	+	+	+	+	?	?	+	+	7
Liu Y 2018	+	+	+	+	+	+	?	?	?	?	6
Xu H 2018	+	+	?	+	+	+	?	?	+	?	6
Chang C 2018	+	?	+	+	?	+	?	?	+	+	6
He Q 2017	+	?	?	+	+	+	?	?	+	?	5
AI Dera H 2017	+	?	?	+	?	+	?	?	+	?	4
Wan D 2016	+	?	+	+	+	?	?	?	+	?	5
Yang HN 2016	+	+	+	+	+	+	?	?	+	?	7
Shi N 2016	+	?	+	+	?	+	?	?	+	+	6
Li Z 2016	+	+	?	+	+	+	?	?	+	?	6
Kizmazoglu C 2015	+	+	?	+	?	+	?	?	+	?	5
Fang LQ 2015	+	+	+	+	+	+	?	?	+	?	7
Li WN 2015	+	?	+	+	?	+	?	?	+	?	5
Girbovan C 2015	+	+	+	+	+	+	?	?	+	?	7
Girbovan C 2015 (2)	+	+	+	+	+	+	?	?	+	?	7
Saleh MC 2014	+	+	?	+	?	?	?	?	+	+	5
Wei H 2014	+	+	+	+	+	+	?	?	+	?	7
Lin Y 2013	+	+	+	+	?	+	?	?	+	+	7
Simão F 2013	+	+	+	+	?	+	?	?	+	?	6
Li Z 2012	+	+	+	+	+	+	?	?	+	?	7
Simão F 2012	+	+	?	+	?	+	?	?	+	?	5
Simão F 2011	+	+	?	+	?	+	?	?	+	?	5
Ren JW 2011	+	+	+	+	+	+	?	?	+	?	7
Li H 2011	+	+	+	+	?	+	?	?	+	?	6
Li C 2010	+	+	+	+	?	+	?	?	+	?	6
Yousuf S 2009	+	+	?	+	?	+	?	?	+	?	5
Della-Morte D 2009	+	+	?	+	?	+	?	?	+	?	5
Tsai SK 2007	+	+	?	+	+	+	?	?	+	?	6
Liu YG 2007	+	?	+	+	?	+	?	?	?	?	4
Shigematsu S 2003	+	?	?	+	?	+	?	?	?	?	3
Sinha K 2002	+	+	?	+	?	+	?	?	+	?	5

(1) peer-reviewed journal; (2) temperature control; (3) animals were randomly allocated; (4) blind established model; (5) blinded outcome assessment; (6) anesthetics used without marked intrinsic neuroprotective properties; (7) animal model (diabetic, advanced age or hypertensive); (8) calculation of sample size; (9) statement of compliance with animal welfare regulations; (10) possible conflicts of interest.

### Meta-analysis of resveratrol for cerebral infarct volume in cerebral I/R injury

**FIGURE 2:**
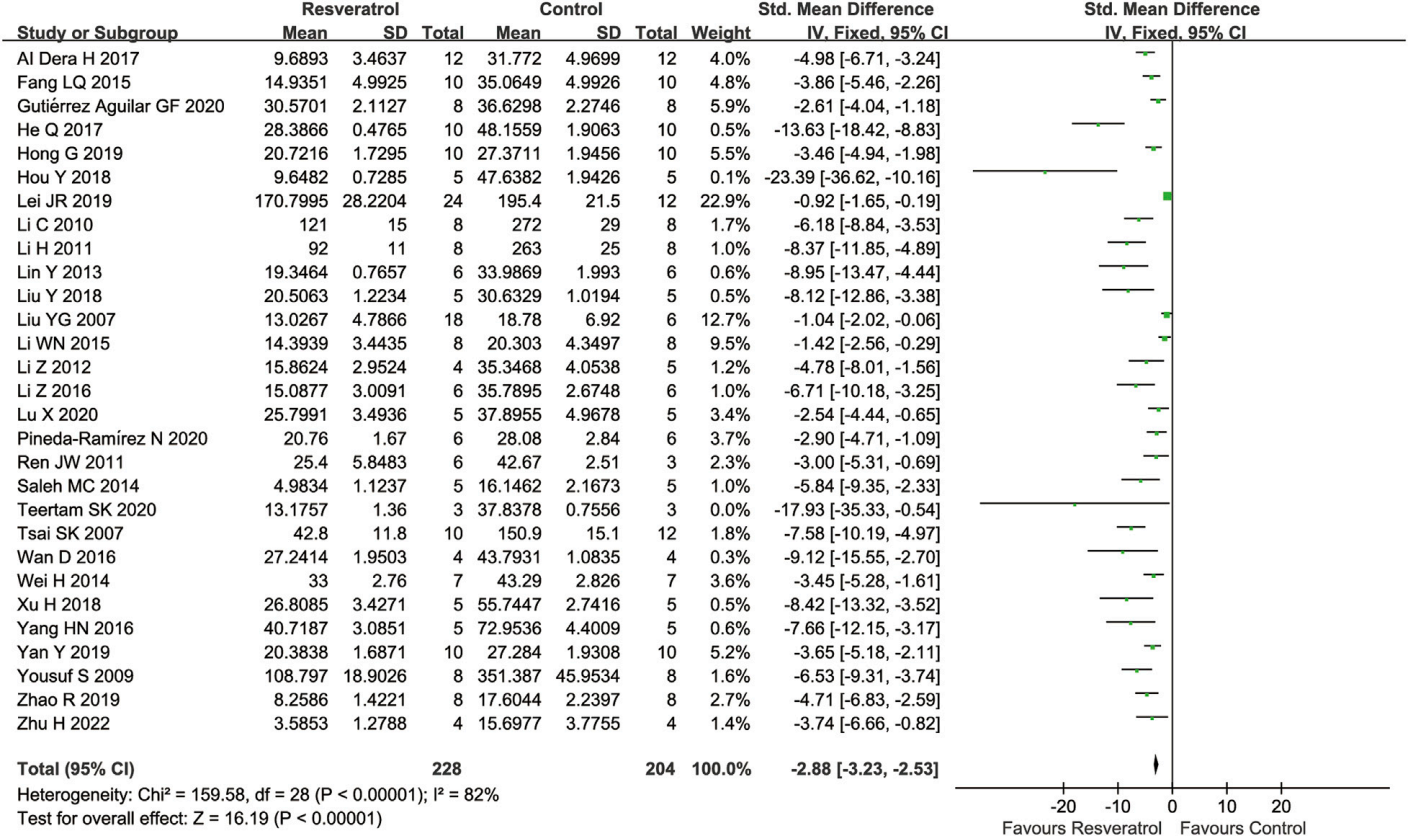
Meta-analysis of resveratrol for cerebral infarct volume in in rats after cerebral I/R injury.

A total of 29 studies assessed the cerebral infarct volume after cerebral IR injury, the result showed that the cerebral infarct volume was substantially decreased after treatment of resveratrol (29 studies, SMD = −2.88 [−3.23 to −2.53], *p* < 0.00001) (Figure 2).

Subgroup analysis suggested that the pooled estimates for improvement of infarct size did not depend on the type of model, species, occlusion time, or timing regimen of pretreatment, but was associated with the route of administration and dosage (Table 3).

**TABLE 3:** Summary of subgroup analysis on cerebral infarct volume.

Pooled estimates	No. of studies	Std. MD (95% CI)	*p* value	Group *p*-value
Model				0.10
MCAO	26	−4.99 [−6.08, −3.90]	*p* < 0.00001	
BCCAO	3	−3.78 [−4.74, −2.83]	*p* < 0.00001	
Species				0.23
SD rats	24	−4.83 [−5.92, −3.73]	*p* < 0.00001	
Long-Evans rats	1	−17.93 [−35.33, −0.54]	*p* = 0.04	
Wistar rats	4	−4.02 [−5.63, −2.40]	*p* = 0.03	
Route of administration				<0.00001
*i.p*	20	−5.18 [−6.46, −3.91]	*p* < 0.00001	
*i.v*	7	−5.06 [−6.72, −3.40]	*p* = 0.003	
*i.g*	1	−1.04 [−2.02, −0.06]	*p* = 0.04	
*ICA*	1	−2.54 [−4.44, −0.65]	*p* = 0.008	
Dosage				0.04
<20 mg/kg	12	−3.07 [−4.32, −1.83]	*p* < 0.00001	
20 mg/kg	8	−3.51 [−5.00, −2.01]	*p* < 0.00001	
30 mg/kg	8	−5.83 [−7.63, −4.04]	*p* < 0.00001	
>30 mg/kg	8	−5.46 [−7.58, −3.34]	*p* < 0.00001	
Administration time				0.58
Before I/R	18	−5.05 [−6.41, −3.70]	*p* < 0.00001	
After I/R	10	−4.50 [−6.15, −2.85]	*p* < 0.00001	
Before and after I/R	2	−3.14 [−6.62, −0.34]	*p* = 0.0008	
Occlusion time				0.04
60 min	3	−10.89 [−16.35, −5.42]	*p* < 0.0001	
90 min	6	−5.12 [−7.57, −2.67]	*p* < 0.0001	
120 min	18	−4.02 [−5.08, −2.97]	*p* < 0.00001	

BCCAO, the bilateral common carotid arteries occlusion; MCAO, middle cerebral artery occlusion; ICA, intra-carotid artery; I/R, ischemia/reperfusion; *i.g.*, intragastric gavage; *i.p.*, intraperitoneal; *i.v.*, intravenous injection; SD, Sprague-Dawley.

Resveratrol is usually administered either by intravenous or intraperitoneal injection in most trials to treat cerebral I/R injury. Compared with the control group, resveratrol significantly reduced the cerebral infarct volume in both intraperitoneal injection (*i.p.*) (20 studies, SMD = −5.18 [−6.46 to −3.91], *p* < 0.00001) and intravenous injection (*i.v.*) (seven studies, SMD = −5.06 [−6.72 to −3.40], *p* = 0.003) in the rat model of cerebral I/R injury. As only few studies have given resveratrol by gavage (*i.g.*) (one study, SMD = −1.04 [−2.02 to −0.06], *p* = 0.04) or intra-carotid artery (ICA) (one study, SMD = −2.54 [−4.44 to −0.65], *p* = 0.008), further studies are needed to confirm these administration regimen.

According to the subgroup analysis, the dose-response effect of resveratrol in the treatment of cerebral I/R injury was observed. The reduction in cerebral infarct volume was more pronounced in studies using doses of 20 mg/kg (eight studies, SMD = −3.51[−5.00 to −2.01], *p* < 0.00001) than in studies using doses of less than 20 mg/kg (12 studies, SMD = −3.07 [−4.32 to −1.83], *p* < 0.00001). Then, a dose of 30 mg/kg (eight studies, SMD = −5.83 [−7.63 to −4.04], *p* < 0.00001) is preferable to 20 mg/kg. Interestingly, when the dose was greater than 30 mg/kg (eight studies, SMD = −5.46 [−7.58 to −3.34], *p* < 0.00001), the cerebral infarct volume could not be further reduced.

A greater reduction in infarct volume in animals given pretreatment with resveratrol (18 studies, SMD = −5.05 [−6.41 to −3.70], *p* < 0.0001) compared with animals given after I/R (10 studies, SMD = −4.40 [−6.15 to −2.85], *p* < 0.0001). Therefore, early administration of resveratrol should be more neuroprotective.

Subgroup analyses showed the shorter occlusion period, the better the effect of resveratrol on reducing cerebral infarction area (*p* = 0.04).The neuroprotective effect of resveratrol is better in 60 min occlusion (three studies, SMD = −10.89 [−16.35 to −5.42], *p* < 0.0001) than 90 min occlusion (six studies, SMD = −5.12 [−7.57 to −2.67], *p* < 0.0001). In addition, resveratrol showed the weakest effect in the 120 min occlusion studies (18 studies, SMD = −4.02 [−5.08 to −2.97], *p* < 0.00001). Accordingly, resveratrol can provide higher improvements on cerebral I/R injury especially for those reveal mild to moderate-severity symptoms.

### Meta-analysis of resveratrol for brain water content in cerebral I/R injury

**FIGURE 3:**
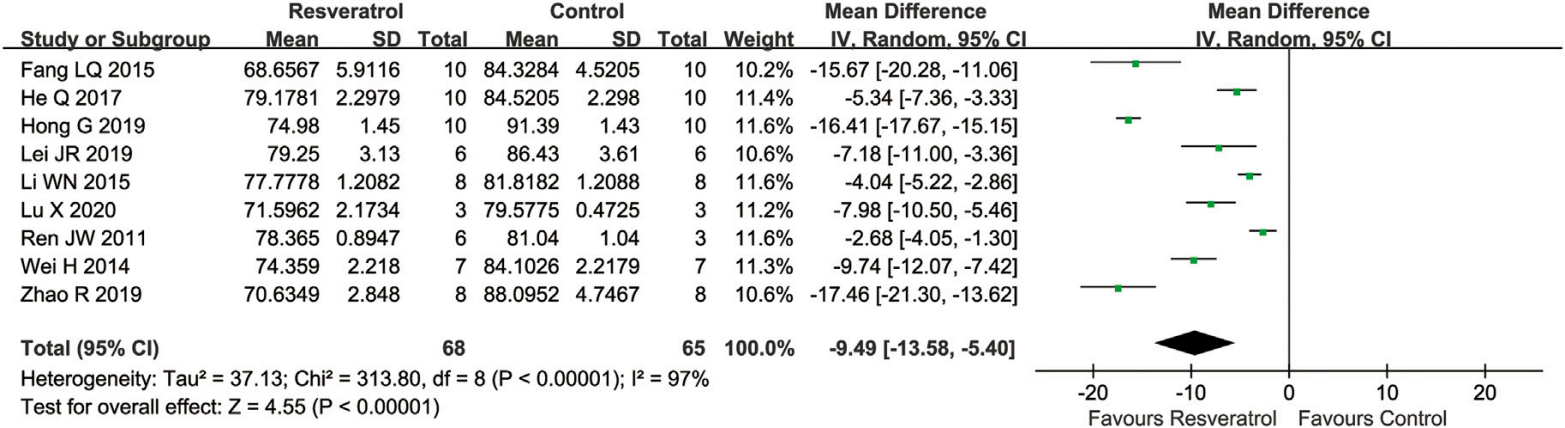
Meta-analysis of resveratrol for cerebral water content in rats after cerebral I/R injury.

Nine studies reported brain water content as outcome. Compared with the control group, the brain water content was significantly reduced by resveratrol (nine studies, MD = −9.49 [−13.58 to −5.40], *p* < 0.00001) (Figure 3).

Subgroup analysis suggested that the pooled estimates for improvement of brain water content was associated with the type of model, the route of administration, dosage, timing regimen of pretreatment, and occlusion time (Table 4). The route of administration is intravenous injection (one study, SMD = −17.46 [−21.30 to −13.62], *p* < 0.00001) rather than intraperitoneal injection. Due to the relatively small number of studies, further studies are needed to confirm this administration regimen, and resveratrol showed the best efficacy of neuroprotection in the 120 min occlusion studies (six study, SMD = −9.92 [−10.71 to −9.13], *p* < 0.00001).

**TABLE 4:** Summary of subgroup analysis on brain water content.

Pooled estimates	No. of studies	Std. MD (95% CI)	*p* value	Group *p*-value
Model				<0.00001
MCAO	8	−5.51 [−6.21, −4.80]	*p* < 0.00001	
BCCAO	1	−16.41 [−17.67, −15.15]	*p* < 0.00001	
Species				
SD rats	9	−8.10 [−8.71, −7.48]	*p* < 0.00001	
Route of administration				<0.00001
*i.p*	7	−7.84 [−8.49, −7.20]	*p* < 0.00001	
*i.v*	1	−17.46 [−21.30, −13.62]	*p* < 0.00001	
*ICA*	1	−7.98 [−10.50, −5.46]	*p* < 0.00001	
Dosage				<0.00001
<20 mg/kg	3	−4.90 [−6.08, −3.71]	*p* < 0.00001	
20 mg/kg	2	−9.83 [−10.70, −8.97]	*p* < 0.00001	
30 mg/kg	2	−4.38 [−5.80, −2.96]	*p* < 0.00001	
>30 mg/kg	3	−7.22 [−8.64, −5.81]	*p* < 0.00001	
Administration time				<0.00001
Before I/R	3	−10.09 [−10.95, −9.22]	*p* < 0.00001	
After I/R	5	−8.49 [−9.80, −7.19]	*p* < 0.00001	
Before and after I/R	1	−4.04 [−5.22, −2.86]	*p* < 0.00001	
Occlusion time				<0.00001
60 min	1	−5.34 [−7.36, −3.33]	*p* < 0.00001	
90 min	2	−5.21 [−6.34, −4.08]	*p* < 0.00001	
120 min	6	−9.92 [−10.71, −9.13]	*p* < 0.00001	

BCCAO, the bilateral common carotid arteries occlusion; MCAO, middle cerebral artery occlusion; ICA, intra-carotid artery; I/R, ischemia/reperfusion; *i.g.*, intragastric gavage; *i.p.*, intraperitoneal; *i.v.*, intravenous injection; SD, Sprague-Dawley.

### Meta-analysis of resveratrol for neurological deficit scores in cerebral I/R injury

**FIGURE 4:**
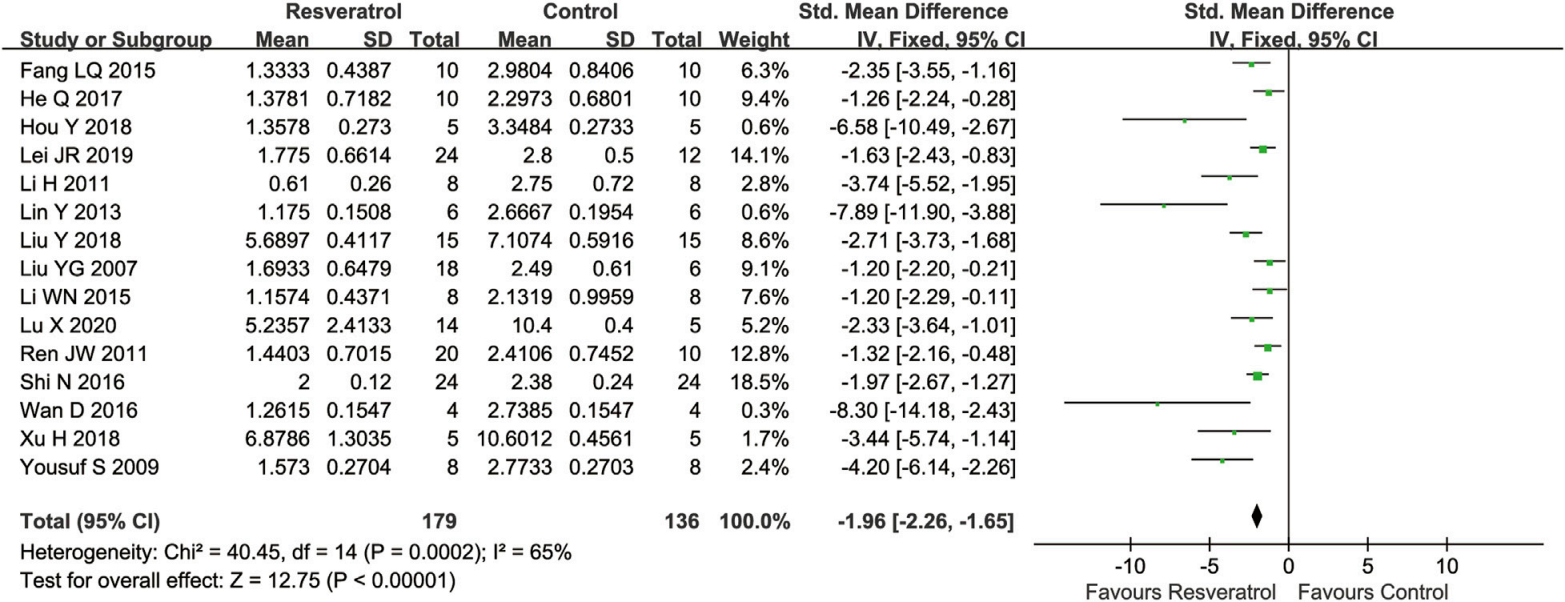
Meta-analysis of resveratrol for neurological deficit scores in rats after cerebral I/R injury.

In terms of functional assessment, 15 studies assessed neurological scores using different scoring systems. A higher neurological function score was associated with greater severity in most of the studies, this meta-analysis showed that resveratrol can significantly improve neurological function in rats (15 studies, SMD = −0.96 [−1.04 to −0.89], *p* < 0.00001) (Figure 4).

Different from the above studies, two studies used other neurological function scoring criteria, namely the 21-Point Behavioral Scale (Normal and maximum Score, 21) and Garcia Score (Wei et al., 2015; Yang et al., 2016), that indicated that the neurological deficit was recovery after treatment with resveratrol (*p* < 0.05). The same results were also indicated in grip test rota rod and closed or open field activity test (Sinha et al., 2002; Zhao et al., 2019), as well as the Morris water maze in these studies (Girbovan et al., 2016; Li et al., 2016; Chang et al., 2018).

Subgroup analysis suggested that the pooled estimates for improvement of neurological deficit scores was associated with the route of administration, dosage, timing regimen of pretreatment, and occlusion time (Table 5). The results of subgroup analysis were similar to those of cerebral infarct volume. 30 mg/kg (four studies, SMD = −1.84 [−2.08 to −1.60], *p* < 0.00001) is the more effective dose. And resveratrol showed the best efficacy of neuroprotection in the 90 min occlusion studies (two study, SMD = −1.33 [−1.66 to −1.01], *p* < 0.00001). Identically, the neurological deficit scores were obviously improved when resveratrol was given after ischemia-reperfusion (four studies, SMD = −1.87 [−2.25 to −1.49], *p* < 0.00001).

**TABLE 5:** Summary of subgroup analysis on neurological deficit scores.

Pooled estimates	No. of studies	Std. MD (95% CI)	*p* value	Group *p*-value
Model				
MCAO	15	−0.96 [−1.04, −0.89]	*p* < 0.00001	
Species				0.07
SD rats	14	−0.95 [−1.02, −0.87]	*p* < 0.00001	
Wistar rats	1	−1.20 [−1.47, −0.94]	*p* < 0.00001	
Route of administration				<0.00001
*i.p*	11	−0.92 [−1.00, −0.85]	*p* < 0.00001	
*i.v*	1	−3.72 [−4.93, −2.51]	*p* < 0.00001	
*i.g*	1	−0.80 [−1.37, −0.22]	*p* = 0.006	
*ICA*	1	−5.16 [−6.48, −3.85]	*p* < 0.00001	
Dosage				<0.00001
<20 mg/kg	7	−1.10 [−1.29, −0.92]	*p* < 0.00001	
20 mg/kg	5	−0.71 [−0.81, −0.62]	*p* < 0.00001	
30 mg/kg	4	−1.84 [−2.08, −1.60]	*p* < 0.00001	
>30 mg/kg	6	−1.62 [−1.76, −1.47]	*p* < 0.00001	
Administration time				<0.0001
Before I/R	10	−0.93 [−1.00, −0.86]	*p* < 0.00001	
After I/R	4	−1.87 [−2.25, −1.49]	*p* < 0.00001	
Before and after I/R	1	−0.97 [−1.73, −0.22]	*p* = 0.01	
Occlusion time				0.008
60 min	1	−0.92 [−1.53, −0.31]	*p* = 0.003	
90 min	2	−1.33 [−1.66, −1.01]	*p* < 0.00001	
120 min	12	−0.95 [−1.02, −0.87]	*p* < 0.00001	

BCCAO, the bilateral common carotid arteries occlusion; MCAO, middle cerebral artery occlusion; ICA, intra-carotid artery; I/R, ischemia/reperfusion; *i.g.*, intragastric gavage; *i.p.*, intraperitoneal; *i.v.*, intravenous injection; SD, Sprague-Dawley.

### Meta-analysis of resveratrol for antioxidative effects in cerebral I/R injury

**FIGURE 5:**
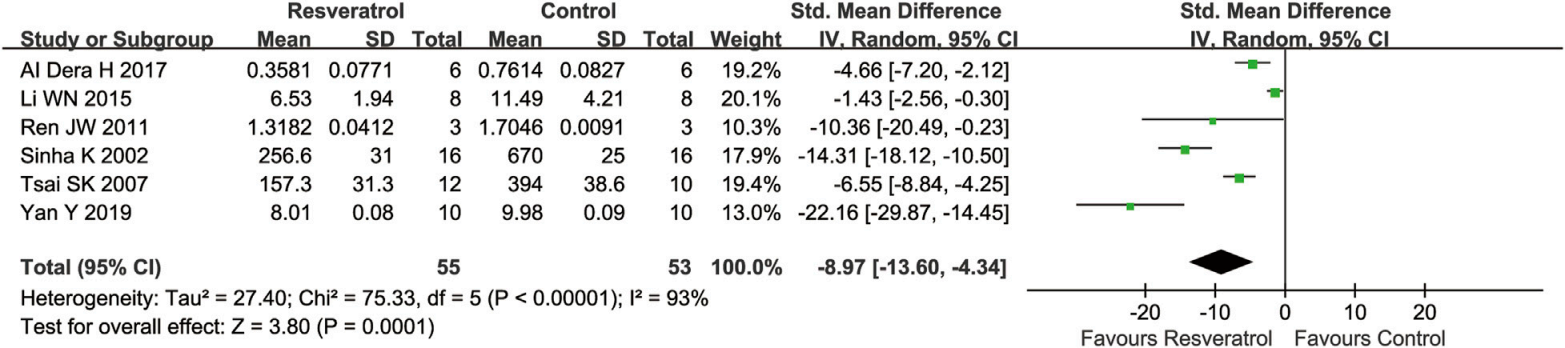
Meta-analysis of resveratrol for MDA in rats after cerebral I/R injury.

**FIGURE 6:**
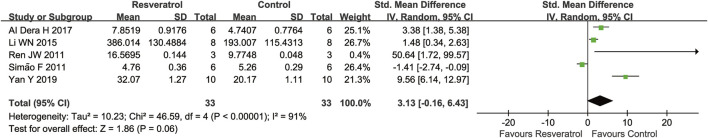
Meta-analysis of resveratrol for SOD in rats after cerebral I/R injury.

Antioxidative effects were measured in seven articles. Six ;studies measured MDA levels after cerebral I/R injury and found that MDA levels were significantly lower in the resveratrol group than the matched group of controls (six studies, SMD = −8.97 [−13.60 to −4.34], *p* = 0.0001) (Figure 5). In accordance with MDA, resveratrol was also associated with a significant amelioration in SOD compared with vehicle treatment (five studies, SMD = 3.13 [−0.16 to 6.43], *p* = 0.06) (Figure 6).

### Publication bias

**FIGURE 7:**
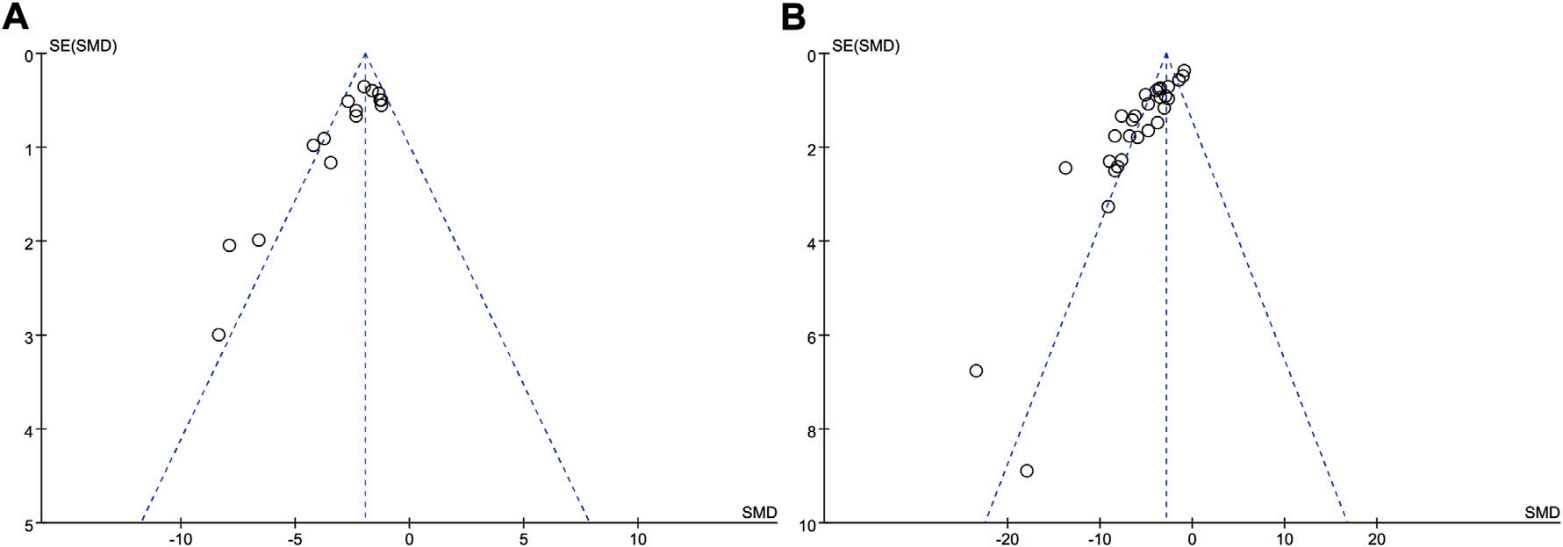
Funnel plots of publication bias for cerebral infarct volume **(A)** and neurological deficit scores **(B)**.

The funnel plots of the cerebral infarct volume were constructed to assess publication bias (Figure 7). Most of the data points in this study were scattered on both sides of the funnel plot, except few far away from the funnel plot, suggesting publication bias was exist at all, and cannot be ignored.

## Discussion

### Summary of main results

In this meta-analysis, we evaluated the neurological recovery effects of resveratrol in rat models of cerebral I/R injury. In agreement with the meta-analysis of Liu et al. (Liu J. et al., 2021), our results showed that resveratrol is associated with a significantly improved infarct size and the neurological scores post-I/R injury *in vivo* animal studies. In addition, cerebral water content in our meta-analysis was well improved. Compared with vehicle groups, resveratrol treatment significantly increased SOD levels and decreased MDA levels in rats after brain I/R injury in the resveratrol groups which provides quantitative evidence for resveratrol in the treatment of cerebral I/R injury in antioxidant stress. Our subgroup analysis suggested that the optimal therapeutic dose is 30 mg/kg, especially for those with mild to moderate-severity cerebral I/R injury.

### Resveratrol dosage and safety

The pharmacokinetics of resveratrol are well understood with high oral absorption but poor bioavailability (Chang et al., 2018). While, the appropriate dose still needs to be concerned. The neuroprotective effect of resveratrol against cerebral I/R injury is dose-dependent in several studies (Liu et al., 2007; Ren et al., 2011; Saleh et al., 2014; Girbovan et al., 2016; Xu et al., 2018; Lu et al., 2020). Our subgroup analyses suggested the optimal dose of resveratrol to treat cerebral I/R injury is 30 mg/kg.

Interestingly, it should be noted that resveratrol at dose over 30 mg/kg will not achieve a more ideal neuroprotective effect, and can’t neglect the possibility of adverse reactions. Brown et al. reported that 5.0 g/day oral dose of resveratrol were well tolerated without any serious consequences, but cause mild to moderate gastrointestinal symptoms (Brown et al., 2010). To evaluate the potential toxicity of resveratrol, Crowell JA, et al. (Crowell et al., 2004) had observed increases in kidney weights and clinically significant renal lesions by administered 3.0 g trans-resveratrol orally per kilogram body weight per day to rats for 4 weeks. Chachay VS., et al. (Chachay et al., 2014) performed a placebo-controlled trial, indicating daily 3.0 g resveratrol treatment did not induce therapeutic benefits in men with established non-alcoholic fatty liver disease. But the significant rise up of alanine aminotranferease and aspartate aminotransferase to week 6 is nevertheless concerning, especially since the long-term effects of resveratrol supplementation were not investigated in the study. Given the minimal toxicity data in rats and the lack of data for systemic toxicity in other species, the potential toxicity of resveratrol was not entirely clear. Hence, more clinical research on resveratrol is needed to determine the minimal effective dose for particular target populations, and pay close attention to possible toxicity on kidney and liver.

In addition, the meta-analysis of Liu et al. (Liu X. et al., 2021) discovered a significant decrease of infarct volume and the neurobehavioral score was achieved in resveratrol sub-groups with a dosage of 20–50 mg/kg among rats and mouse, then we made this dose accurate to 30 mg/kg in rats. According to the body surface area method, the conversion coefficient between different animals and humans is different, the dosage given to mice is about 12.3 times that of humans, rats about 6.2 times that of humans, simple merging of mouse and rat data might was inappropriate, then our reseult also needs be proved by *in vivo* experiments (Guidance for Industry 2005).

### Potential therapeutic mechanisms

**FIGURE 8:**
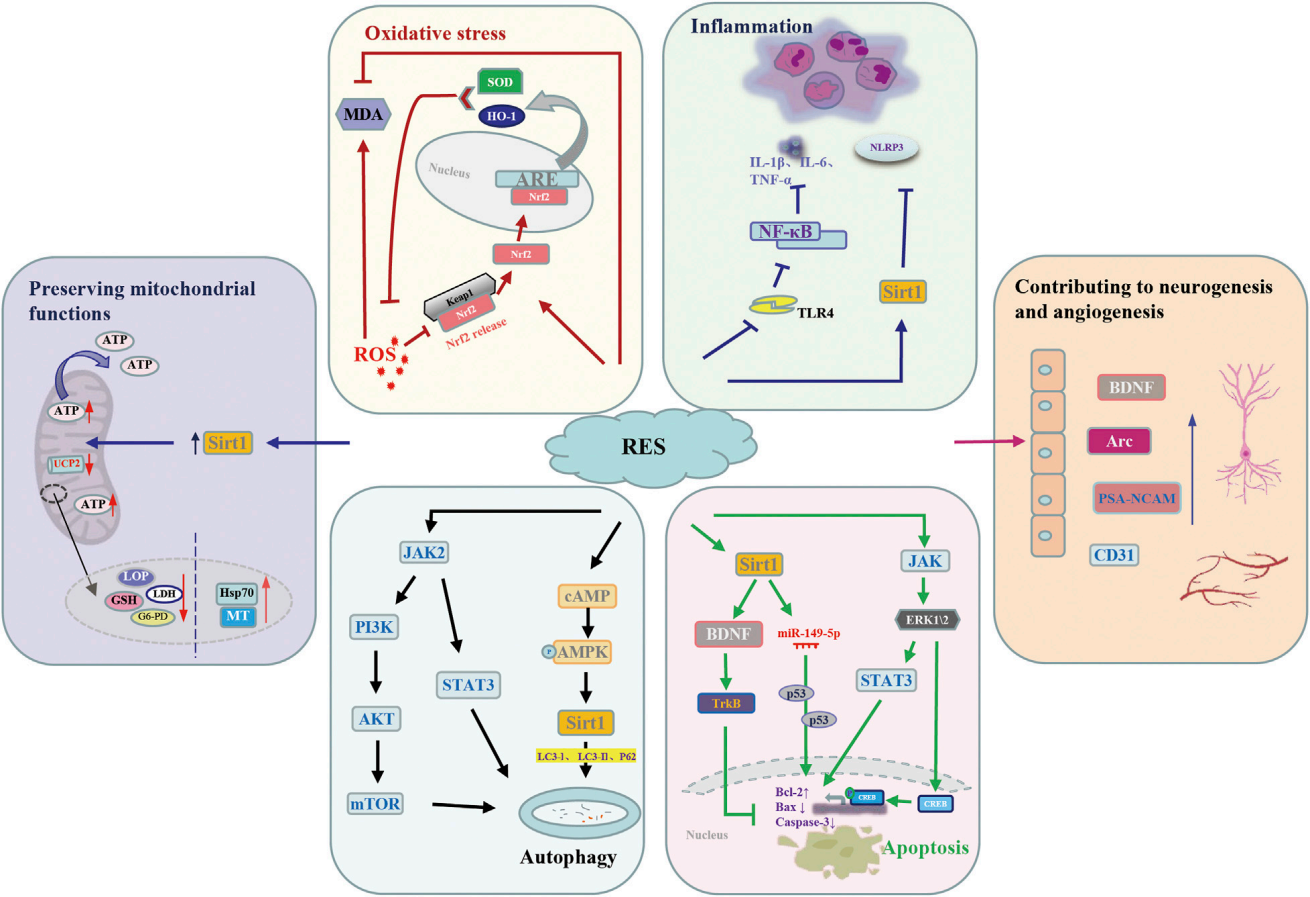
Potential molecular mechanisms underlying the neuroprotective action of resveratrol against cerebral I/R injury. AMPK, AMP-activated protein kinase; Arc, Activity-regulated cytoskeleton-associated; ARE, antioxidant response element; Bax, bcl-2-associated x; Bcl-2, b-cell lymphoma-2; BDNF, brain derived-nerve neurotrophic factor; CD31, platelet and endothelial cell adhesion molecule 1; CREB, cyclic-AMP response element binding protein; ERK, extracellular signal-regulated kinases; G6-PD, glucose 6-phosphate dehydrogenase; GSH, glutathione; HO-1, heme oxygenase 1; Hsp70, heatshockprotein70; IL-1β, interleukin-1beta; IL-6, serum interleukin-6; JAK, janus kinases; Keap l, Kelch-like ECH-associated protein 1; LC3, light chain 3; LDH, lactate dehydrogenase; LPO, lipid peroxidation; MDA, malondialdehyde; MT, metallothionein; mTOR, mammalian target of rapamycin; NF-κB, nuclear factor-κB; NLRP3, NOD-like receptor thermal protein domain associated protein 3; Nrf2, nuclear factor erythroid 2-related factor 2; p62, sequestosome-1; PI3K, phosphoinositide 3-kinase; PSA-NCAM, Polysialylated-Neural Cell Adhesion Molecule; RES, resveratrol; ROS, reactive oxygen species; Sirt1, silent mating type information regulation 2 homolog 1; STAT3, signal transducers and activators of transcription 3; SOD, superoxide dismutase; TrkB, tyrosine kinase receptor B; TLR4, Toll-like receptor 4; TNF-α, tumor necrosis factor-α; UCP2, uncoupling protein 2.

It is a common phenomenon that oxidative stress injury, inflammatory reaction, and apoptosis result in brain damage after I/R injury. Large numbers studies demonstrated that resveratrol, an anti-inflammatory and antioxidant substance, have exerts significant neuroprotective effects. Even though, the pathological mechanism of brain I/R injury remains unclear, several signaling pathways related to antiapoptotic capacity, anti-inflammation, antioxidative stress and autophagy of resveratrol were involved in the process. In addition, resveratrol can play a neuroprotective role by maintaining mitochondrial function and promoting neurogenesis and angiogenesis (Table 6 and Figure 8).

**TABLE 6:** The proposed molecular and cellular mechanism of the neuroprotective effect of resveratrol.

Study	Mechanism	Outcome
Zhu H 2022	Apoptosis	Inhibit ferroptosis induced by OGD/R and ferroptosis inducers
Gutiérrez Aguilar GF 2020	—	Activation of AMPK signaling, downregulation GLUT3 expression
Teertam SK 2020	Apoptosis	Sirt1, p53, caspase-3; Activation of Sirt1/miR-149-5p/p53 axis; downregulation caspase-3
Lu X 2020	Oxidative stress, apoptosis, contributing to neurogenesis and angiogenesis	MDA, Bax, cleaved caspase-3, Bcl-2; Enhance expression of BDNF
Pineda-Ramírez N 2020	Autophagy	AMPK, Beclin 1, LC-I, LC-II, p62; Activation of AMPK/autophagy pathway
Hong G 2019	Apoptosis	YAP, TAZ; Activation of the Hippo/YAP/TAZ signaling pathway, improve BBB breakdown
Zhao R 2019	Inflammation, apoptosis	1Iba-1 and MPO, Bcl-2, Bcl-xl, Bad and Bax; Downregulation of TGF-β-mediated ERK pathway, improve BBB breakdown
Lei JR 2019	Inflammation	MPO, TLR4, NF-κB, p65, COX-2, MMP9, TNF-α and IL-1β; Downregulation of TLR4 signaling pathway
Yan Y 2019	Inflammation, oxidative stress, apoptosis	IL-6, TNF-α, SOD, ROS, MDA, Bcl-2, Bax; modulation of the BMP-4/ROS/COX-2 pathway
Serra MP 2019	contributing to neurogenesis and angiogenesis	BDNF, TrkB, PSA-NCAM, and Arc
Hou Y 2018	Apoptosis	Bcl-2, Bax, caspase-3; Activation of the JAK2/STAT3/PI3K/AKT/mTOR pathway
Liu Y 2018	Apoptosis, Inflammation, autophagy	Bcl-2, Bax, caspase-3, IL-1β, and Beclin-1, LC3II/LC3I
Xu H 2018	Oxidative stress	MDA
Chang C 2018	Oxidative stress, inflammation, apoptosis	SOD, MDA, TNF-α and IL-6, Bcl-2, Bax; Regulation of JAK/ERK/STAT signaling pathway
He Q 2017	Inflammation, autophagy	NLRP3 inflammasome, caspase-1, IL-1β, and IL-18, Sirt1, LC3B-II, LC3B-I, p62; inhibition of NLRP3 inflammasome activation through Sirt1-dependent autophagy induction
AI Dera H 2017	Oxidative stress, apoptosis, inflammation	SOD, MDA, CAT, GPx, OPN, iNOS, Bax, IL-1β, KC, MIP-2, ICAM-1
Wan D 2016	Autophagy	Inhibition of PDEs and activation of the cAMP/AMPK/SIRT1 pathway
Yang HN 2016	Inflammation	IL-6, TNF-α, Tregs
Shi N 2016	Apoptosis	Activation of the Sirt1/BDNF/TrkB pathway
Li Z 2016	Apoptosis	Regulation of the NMDA receptor-mediated ERK1/2-CREB signaling pathway
Kizmazoglu C 2015	Apoptosis	Bcl-2, p53
Fang LQ 2015	Apoptosis, inflammation	Caspase-3, cleaved caspase3, Bcl-2, Bax, and β-actin, MPO, TNF-α
Li WN 2015	Oxidative stress	MDA, SOD, iNOS, Brain edema (reduce AQP4 expression)
Girbovan C 2015 (1)	contributing to neurogenesis and angiogenesis	Neurogenesis, angiogenesis, and corticosterone secretion
Girbovan C 2015 (2)	contributing to neurogenesis and angiogenesis	Downregulates type-1 glutamate transporter expression and microglia activation
Saleh MC 2014	—	—
Wei H 2014	—	BBB Integrity, MMP-9 and TIMP-1, regulation of the TIMP-1 signal
Lin Y 2013	—	Regulation of TRPC6/CREB pathways
Simão F 2013	—	Lipid content (ganglioside content)
Li Z 2012	Apoptosis	Bcl-2, Bax
Simão F 2012	Autophagy	Activation of the PI3-K⁄Akt pathway; Downregulation of GSK-3b and CREB
Simão F 2011	Oxidative stress	ROS, NO, Na+, K+-ATPase, SOD, GPx
Ren JW 2011	Oxidative stress, Apoptosis	MDA, SOD, caspase-3; Activation of the Nrf2/ARE/HO-1 signaling pathway
Li H 2011	Oxidative stress	Brain energy metabolism (Glucose, lactate, ATP, EC, hypoxanthine, xanthine); MDA, XO
Li C 2010	—	Regulation of neurotransmitter and neuromodulator (Arg, Lys, Trp, Leu, Phe, Gln, GABA, D-Ser, L-Ser, Ala, Tau, Gly, DA, PEA, Glu, and Asp)
Yousuf S 2009	Preserving mitochondrial functions	GSH, G6-PD, LDH, LPO, H_2_O_2_,MT, Hsp70
Della-Morte D 2009	Preserving mitochondrial functions	SIRT1, UCP2, membrane potential, respiration, and the itochondrial ATP synthesis efficiency
Tsai SK 2007	Oxidative stress	MDA, NO
Liu YG 2007	Inflammation	TNF-α, MPO, IL-6, IL-1β
Shigematsu S 2003	Inflammation, Oxidative stress	—
Sinha K 2002	Oxidative stress	glutathione, MDA

AQP4, aquaporin 4; AMPK, AMP-activated protein kinase; ARE, antioxidant response element; ATP, adenosine triphosphate; Bax, bcl-2-associated x; BBB, blood-brain barrier; Bcl-2, b-cell lymphoma-2; BDNF, brain derived-nerve neurotrophic factor; BMP-4, bone morphogenetic protein-4; CAT, catalase; CREB, cyclic-AMP response element binding protein; COX-2, cyclooxygenase-2; ERK, extracellular signal-regulated kinases; G6-PD, glucose 6-phosphate dehydrogenase; GLUT3, glucose transporter 3; GPx, glutathione peroxidase; GSH, glutathione; H_2_O_2_, hydrogen peroxide; HE, histopathological estimation; HO-1, heme oxygenase 1; Hsp70, heat stress protein; 1Iba-1, ionized calcium binding adaptor molecule 1; IL-1β, interleukin-1beta; IL-6, serum interleukin-6; JAK, janus kinases; Keap l, Kelch-like ECH-associated protein 1; LDH, lactate dehydrogenase; LC3, light chain 3; MDA, malondialdehyde; MT, metallothionein; MMP-9, matrix metalloproteinase-9; MPO, myelo peroxidase; mTOR, mammalian target of rapamycin; NF-κB, nuclear factor-κB; NLRP3, NOD-like receptor thermal protein domain associated protein 3; Nrf2, nuclear factor erythroid 2-related factor 2; P-AMPK, phosphorylation of adenosine-monophosphate-activated protein kinase; PDEs, phosphodiesterase; p62, sequestosome-1; PI3K, phosphoinositide 3-kinase; ROS, reactive oxygen species; Sirt1, silent mating type information regulation 2 homolog 1; STAT3, signal transducers and activators of transcription 3; SOD, superoxide dismutase; TTLR4, Toll-like receptor 4; TNF-α, tumor necrosis factor-α; rkB, tyrosine kinase receptor B; UCP2, mitochondrial uncoupling protein 2; XO, xanthine oxidase.

#### Antioxidative stress

Oxidative stress is an important part of the complex mechanism of cerebral I/R injury. MDA, the principal product of lipid peroxidation, is regarded as a biomarker for oxidative stress after cerebral I/R injury. Great interest has been focused on SOD its significant reactive oxygen species (ROS) scavenging ability. It is necessary to find a substance that can exert an antioxidant effect in the brain tissue. Fortunately, due to its significant SOD activation ability, the antioxidant effect of resveratrol in cerebral I/R injury has been proven (Tsai et al., 2007; Li et al., 2011). At the same time, the level of MDA was downregulated by resveratrol pretreatment (Yan et al., 2019). Activation of the nuclear factor erythroid 2-related factor 2 (Nrf2)/antioxidant response element (ARE) signaling pathway has protective effect on cerebral I/R injury. Normally, Nrf2 interacts with Kelch-like ECH-associated protein 1 (Keap l) to form the Keap-1-Nrf2 complex and restrict Nrf2-mediated gene expression. When Nrf2/ARE signaling pathway activated, the Keap-1-Nrf2 complex dissociates, and Nrf2 is translocated into the nucleus to bind to ARE, activate expression of heme oxygenase-1 (HO-1) and SOD to attenuate cellular oxidative stress. Based on this, resveratrol can ameliorate oxidative stress injury caused by cerebral I/R by up-regulating Nrf2 and HO-1 expression (Ren et al., 2011).

#### Anti-inflammation

Growing evidence demonstrated that inflammation has a significant impact on ischemic brain injury in animal studies or patients with ischemic stroke. Historically, the role of resveratrol in anti-inflammation effect is beyond debate. It has been shown that resveratrol can not only increased the production of T regulatory cells (Tregs) in the ischemic hemisphere but also regulate the elevation of IL-6 and TNF-α levels in peripheral blood after 24 h cerebral I/R injury (Yang et al., 2016). Studies have shown that NOD-like receptor family, pyrin domain-containing 3 (NLRP3) inflammasome and its downstream cytokines play a key role in the occurrence of brain I/R injury. Interestingly, under the intervention of Sirt1, resveratrol could inhibit NLRP3 inflammasome activation (He et al., 2017). According to report, resveratrol could alleviate inflammatory response by downregulating Toll-like receptor 4/nuclear factor-κB (TLR4/NF-κB) signaling pathway, and thus alleviate ischemic brain injury induced by inflammation (Lei et al., 2019).

#### Anti-apoptotic capacity

Apoptosis increased with the occurrence of ischemia-reperfusion. Resveratrol was thought to alleviate nerve injury by downregulating bcl-2-associated x (Bax) and caspase-3 (Fang et al., 2015; Liu et al., 2018; Lu et al., 2020), which considered as the executioner caspase for its role in the destruction of cellular structures. Recent studies demonstrated that the neuroprotective mechanisms of resveratrol may be attributed to its involved in a variety of signaling pathways (Chang et al., 2018; Zhao et al., 2019; Teertam et al., 2020). It has been concluded that sirtuin1 (Sirt1) can be activated by resveratrol treatment, and subsequent upregulation of Mir-149-5p reduces the loss of neurons that may bind to p53, thereby reducing the activity of caspase-3 (Teertam et al., 2020). The expression levels of brain derived-nerve neurotrophic factor (BDNF) and tyrosine kinase receptor B (TrkB) in resveratrol group were significantly increased, which may be related to the upregulation of BDNF/TrkB signaling pathway (Shi et al., 2016; Serra et al., 2019). Furthermore, upregulation of the janus kinases (JAK), extracellular signal-regulated kinases (ERK) and signal transducers and activators of transcription (STAT) pathway or extracellular signal regulated kinase (ERK) and cyclic-AMP response element binding protein (CREB) signaling might participates in neuroprotection of resveratrol (Li et al., 2016; Chang et al., 2018).

#### Autophagy

Accumulating evidence shows that a variety of cell types in the brain after ischemic stroke will activate autophagy, which plays an important homeostasis role in regulating cell survival. Resveratrol is considered as an interesting target in cerebral I/R injury research due to its regulation of autophagy (Liu et al., 2018). In spite of this, the exact mechanism of how resveratrol plays a neuroprotective role in cerebral I/R injury by regulating autophagy has not been clarified. Studies have shown that the AMP-activated protein kinase (AMPK) activation is involved in the neuroprotective effects of resveratrol (Gutierrez Aguilar et al., 2020). Furthermore, they demonstrate that resveratrol increases AMPK activity and drives autophagy (Pineda-Ramirez et al., 2020). Sirt1, one of the targets of resveratrol, can be activated to exert neuroprotective effects, including autophagy regulation (He et al., 2017). By up-regulating the phosphorylation levels of Akt and the mammalian target of rapamycin (mTOR) proteins, the phosphoinositide 3-kinase (PI3K)/Akt/mTOR signaling pathway is activated and autophagy is mediated. Resveratrol considerably improved neurological function and inhibits the apoptosis of neuron cells after stroke, which is partially induced by activation of the JAK2/STAT3/PI3K/AKT/mTOR signaling pathway (Hou et al., 2018).

#### Preserving mitochondrial functions

In the process of cerebral I/R injury, oxidative stress, calcium overload, inflammatory response and other factors can easily lead to neuronal mitochondrial imbalance. Resveratrol plays a neuroprotective role by regulating mitochondrial dysfunction during cerebral ischemia. Compared with MCAO group, ATP level in MCAO + resveratrol group was significantly increased by resveratrol treatment (*p* < 0.01) (Yousuf et al., 2009). Della-Morte D et al. demonstrated that resveratrol pretreatment exerts neuroprotective effects by reducing uncoupling protein 2 (UCP2) levels and increasing the efficiency of mitochondrial ATP synthesis (Della-Morte et al., 2009).

#### Contributing to neurogenesis and angiogenesis

Angiogenesis in the injured CA1 region was increased in resveratrol-treated rats in a dose-dependent manner, which indicated the neuroprotective effect of resveratrol and its ability to upregulate angiogenesis (Girbovan et al., 2015). Its ability to regulate angiogenic processes may be associated with the ability to activate silent information regulator 1 (SIRT1), which is highly expressed in the vascular endothelium during neovascularization (Yang et al., 2013). Resveratrol also can promotes neurogenesis by enhancing the expression of brain-derived neurotrophic factor (BDNF) (Lu et al., 2020), which is an important regulator of neuronal network plasticity, nerve regeneration and neuroprotection (Brigadski and Leßmann, 2020). Resveratrol treatment is effective in inducing neuronal plasticity by increasing BDNF, PSA-NCAM and Arc in the frontal cortex of BCCAO/R rats (Serra et al., 2019). Further studies are needed to assess whether the RVT-induced enhancement of plasticity-related molecules is directly related to the altered dendritic morphology of rat prefrontal cortex neurons.

## Limitations

Several limitations should be considered, first, despite the quality of the included studies was acceptable, there are still some shortcomings. For example, no studies included here described sample-size calculation and allocation concealment. Twenty studies reported randomization, but did not describe what specific adopted random method they used. More than half of the studies did not report blind evaluation of results. There are only a few studies were clearly proposed to screen according to neurological function score. There is also a lack of detailed access to experimental data. Due to the above reasons, the quality of the included literatures was not high enough. We therefore suggest that in future animal model studies, blinded outcome evaluation, use of target animal models (hypertension, advanced age, and diabetes), calculation of sample size, declaration of compliance with animal welfare regulations, possible conflicts of interest, and detailed data release should be considered. Second, it is worth noting that our study contains signs of publication bias, we can see all the published papers that assess infarct volume or neurological deficit score showed a positive (protective) result, which had to make us question the reliability of the results. Negative results may not be available because they may not be favored by the journal. In general, the existence of publication bias clearly undermines our confidence in this group of studies. Therefore, we believe that it is necessary to establish an authoritative registration website for animal experiments, through which some negative or unpublished experiments can be searched for analysis, and thus reduce the risk of publication bias. Due to the quality of the included literature and publication bias, as well as other unknown risk of bias, the conclusion cannot be substantiated based on the data provided, it need to be further verified through exploration of dose effect relationship, or delay administration or not.

## Conclusion

Our current findings suggest that resveratrol might have a distinct neuroprotective effect in rat, the recommended dosage is 30 mg/kg, especially for those with mild to moderate-severity cerebral I/R injury, then it need to be further verified through exploration of dose effect relationship, or delay administration or not. Thus high quality *in vivo* experiments were still encouraged to research the neuroprotective function of resveratrol. As neurological function scores were performed 24 h after I/R in most of the studies, therefore, the long-term efficacy of resveratrol still needs to be further confirmed.

## Data Availability

The original contributions presented in the study are included in the article/Supplementary Material, further inquiries can be directed to the corresponding authors.
